# Advanced Control over Cell-Material Interfaces

**DOI:** 10.3390/polym9120704

**Published:** 2017-12-12

**Authors:** João F. Mano, Insung S. Choi

**Affiliations:** 1 Department of Chemistry, CICECO—Aveiro Institute of Materials, University of Aveiro, 3810-193 Aveiro, Portugal; 2 Center for Cell-Encapsulation Research, Department of Chemistry, KAIST, Daejeon 34141, Korea

Cells in vivo sense and respond to signals from their environment (e.g., other cells and extracellular matrices) for their orchestrated behaviour and function. These signals exist in various forms, including biochemical, electrochemical, structural, and mechanical signals. In vitro mimicry of juxtacrine interactions has been achieved mainly through the use of polymers in regenerative medicine and cell therapy. The notion of scaffolds, in particular, has been closely linked with three-dimensional (3D) or semi-3D structures onto which or into which the cells grow and proliferate. The primary function of scaffolds was initially structural support, providing physical sites for cell adhesion and growth in three dimensions. However, recent scientific and technological developments have advanced to enable the manipulation of cell behaviour and function, ultimately controlling cell fate by the use of specific polymers, including supramolecular hydrogels.

The purpose of this Special Issue is to highlight the recent achievements in the use of polymers as cell scaffolds on a broad scale, examining not only the use of polymers as cell culture scaffolds, but also including polymer-based approaches for controlling interfacial interactions of cells in vitro. Herein are proposed new biomaterials as cell supports, such as injectable copolymers or gelatin-based hydrogels for 3D bioprinting. In other reports, the incorporation of inorganic or organic fillers into polymer matrices has been attempted to improve the structure and function of biomaterials, exemplified by the reinforcement of poly(ε-caprolactone) (PCL) with calcium-containing mesoporous particles or tricalcium phosphate; poly(lactic-*co*-glycolic) (PLGA) with biphasic calcium phosphate or nanohydroxyapatite; poly(lactic acid) (PLA) with Fe_3_O_4_ nanoparticles; and poly(3-hydroxybutyrate-*co*-3-hydroxyvalerate) with calcium silicate. Natural polymers also have been combined with distinct fillers: Human mesenchymal stem cells (hMSCs) differentiation could be regulated by the addition of hydroxyapatite to chitosan-based scaffolds; and alginate hydrogels may be reinforced with cellulose nanofibrils. More complex systems were envisaged for some specific cases, such as the infiltration of photo-crosslinkable hyaluronic acid hydrogels in fused deposition-manufactured composite scaffolds of hydroxyapatite and poly(ethylene glycol)-*b*-PCL.

Scaffold geometries beyond the conventional porous 3D supports are also explored in this Special Issue. Membranes made of biodegradable or natural polymers are proposed, including multilayered free-standing films made of polysaccharides as a potential platform for the formation of human adipose-derived stem cell aggregates. Quasi-3D PCL supports were prepared by electrospinning for breast cancer cell culture. In another study, tubular PLGA-based structures were developed to be used as scaffolds for small-diameter vascular tissue engineering, and micro-needle cuffs were investigated for perivascular drug delivery. Surface modifications, another fundamental approach to enhance cell interactions to scaffolds, are also presented: catechol-conjugated dextran is reported as a surface coating material, showing antiplatelet capabilities; plasma treatment was studied in the modification of polyethylene terephthalate. In another study, atomic force microscopy is suggested as a powerful method for characterizing the scaffold surfaces. The manipulation of individual cells is suggested as an emerging technique in cell scaffold research: the 3D single-cell assembly allowed for structure formation without the need for artificial scaffolds; cells could be included as individual entities or in the form of aggregates into hydrogels, exhibiting distinct behaviour in cartilaginous tissue formation. In another paper, the coating of individual red blood cells was shown to radically change their behaviour, including immunogenicity.

In short, this Special Issue deals with important recent advances in cell-polymer interactions, ranging from the design, fabrication, and modification of cell scaffolds to single-cell manipulation, by contribution from experts in their repective fields. We believe that this issue conveys useful information to scientists and engineers in various fields including polymer scientists and biomedical engineers.

